# Simulation of Gold Nanoparticle Transport during MHD Electroosmotic Flow in a Peristaltic Micro-Channel for Biomedical Treatment

**DOI:** 10.3390/mi13030374

**Published:** 2022-02-26

**Authors:** Muneerah Al Nuwairan, Basma Souayeh

**Affiliations:** 1 Department of Mathematics and Statistics, College of Science, King Faisal University, P.O. Box 400, Al-Ahsa 31982, Saudi Arabia; msalnuwairan@kfu.edu.sa; 2 Department of Physics, College of Science, King Faisal University, P.O. Box 400, Al-Ahsa 31982, Saudi Arabia; 3 Laboratory of Fluid Mechanics, Physics Department, Faculty of Sciences of Tunis, University of Tunis El Manar, 2092 Tunis, Tunisia

**Keywords:** bioconvection, activation energy, gyrotactic microorganisms, gold nanoparticles, physiological flow, electroosmosis

## Abstract

The study of gold nanoparticles (AuNPs) in the blood flow has emerged as an area of interest for numerous researchers, due to its many biomedical applications, such as cancer radiotherapy, DNA and antigens, drug and gene delivery, in vitro evaluation, optical bioimaging, radio sensitization and laser phototherapy of cancer cells and tumors. Gold nanoparticles can be amalgamated in various shapes and sizes. Due to this reason, gold nanoparticles can be diffused efficiently, target the diseased cells and destroy them. The current work studies the effect of gold nanoparticles of different shapes on the electro-magneto-hydrodynamic (EMHD) peristaltic propulsion of blood in a micro-channel under various effects, such as activation energy, bioconvection, radiation and gyrotactic microorganisms. Four kinds of nanoparticle shapes, namely bricks, cylinders and platelets, are considered. The governing equations are simplified under the approximations of low Reynolds number (LRN), long wavelength (LWL) and Debye–Hückel linearization (DHL). The numerical solutions for the non-dimensional equations are solved using the computational software MATLAB with the help of the bvp4c function. The influences of different physical parameters on the flow and thermal characteristics are computed through pictorial interpretations.

## 1. Introduction

Recent developments in nanotechnology have motivated the creation of various nanoparticles [1,2,3,4]. Among the existing nanoparticles, metallic nanoparticles have been used widely in biomedical treatments and, among them, AuNPs attract extreme attention, due to their inherent characteristics, such as surface plasmon resonance (SPR), and their physicochemical, electronic and optical fields, which can be easily modified by converting the particle characterizations, such as environment, aspect ratio, size and shape. This leads to the provision of extreme functionalization properties, of use in many applications in diverse categories of biomedicine, for instance, in imaging, targeted drug delivery, sensing, bioimaging, photodynamic and photothermal therapy. Gold nanoparticles have been greatly used for diagnostics of disease, therapeutics [5], tumor necrosis factor, transmission electron microscopy, scanning electron microscopy, polyethylenglycol and plasmonic photothermal therapy [6]. In the last few decades, many researchers have shown the various applications of gold nanoparticles in diverse areas. For instance, Elahi et al. [7] provided a short review on the various applications of gold nanoparticles. In their study, they have concluded that gold nanoparticles have many applications in biomedicine, such as oncological diseases, certain skin or infectious diseases, hyperthermia, X-ray imaging and photoacoustic imaging. Daraee et al. [8] proved the AuNPs can play an excellent role in solving the problems of bioimaging (optical coherence tomography, confocal laser microscopy and resonance scattering dark-field microscopy). Bansal et al. [9] provided a review on the achievements in and the importance of AuNPs in the biomedical field. AuNPs are widely used in nanoelectronics, as has been shown by Homberger and Simon [10]. In light of the aforementioned reviews and experimental works, more recently, investigators have started working on nanofluid flows suspended by gold nanoparticles from a biological point of view. Mekheimer et al. [11] provided a theoretical study on the peristaltic propulsion of blood flow with gold nanoparticles as a third-grade nanofluid in a catheter, which is applicable in cancer therapy. Koriko et al. [12] presented MATLAB bvp4c solutions for the blood–gold Carreau nanoliquid flow. Ellahi et al. [13] used the homotopy perturbation method to study the blood flow of a nanofluid in composite stenosed arteries. Riaz et al. [14] studied the Jeffrey nanofluid flow in two eccentric asymmetric annuli with the help of the homotopy perturbation method. Elnaqeeb et al. [15] discussed the propulsion of gold nanoparticles in the blood in overlapped stenosis.

It is well known that the magnetic field can be used to treat various diseases. The Arabian physician Avicenna used magnets to treat diseases of the liver in about 1000 A.D. The Persian physician Abbas wrote a book named “Perfect Book of the Art of Medicine” and claimed that magnetism can cure gout and spasms. Later, the Swiss physician Paracelsus used magnets to treat hernias, dropsy and jaundice [16]. Magnetic fields can cure many ailments and is used in the hyperthermia treatment of cancer, as an MRI contrast agent, drug delivery method, in magnetic bio-separation and nervous disorders [17]. Roth [18] provided a review on the applications of magnetic forces in medicine, such as magnetic resonance imaging, magneto-acoustic tomography, Hall effect imaging and magneto-acoustic imaging. Zablotskii et al. [19] proved that magnetic fields affect cell life and discussed their many applications in targeted stem cell delivery, cell therapy, cell biology, and nanomedicine. In view of the aforementioned applications, many researchers have started working on magnetic fields towards the achievement of physiological vessels. Eldabe et al. [20] used the differential transform method to investigate the MHD Carreau nanofluid flow in a peristaltic channel suspended by gold nanoparticles, which was useful in destroying cancer cells. Abdelsalam and Bhatti [21] provided the analytical results of the MHD peristaltic nanofluid flow with suspended gold nanoparticles and concluded that their findings may be useful in in eradicating tumor cells. Akram et al. [22] obtained the exact solutions for the nanofluid flow through a peristaltic channel under a uniform magnetic field. El-Dabe et al. [23] utilized a numerical technique, named the Runge–Kutta–Merson method, to discuss the motion of a nanofluid during inclined peristalsis. Devaki et al. [24] discussed the MHD nanofluid flow in an artery with mild stenosis and concluded that their findings may be helpful in destroying of the stencils. Reddy and Makinde [25] used the Runge–Kutta–Fehlberg method to depict the results of MHD Jeffrey nanofluid motion in an asymmetric channel.

In the last few decades, the level of electromagnetic fields (EMFs) of natural and man-made origin has continuously increased, due to their wide variety of applications in diverse research fields, such as wound healing, cartilage/bone repair, pain control, inhabitation of cancer growth, plastic surgery, electro-chemotherapy, gene therapy, non-thermal ablation, tendon injuries [26]. Ryan et al. [27] provided an experimental study on electric field simulation for tissue engineering applications. Cen and Chen [28] presented electric field applications found in pulsed electric field treatments in vitro and in vivo. There are other applications that have been shown in transcranial magnetic stimulation and nerve electrophysiology with respect to electric and magnetic fields [29]. Moatimid et al. [30] discussed the influences of electroosmosis on nanofluid flow in a peristaltic micro-channel and that are useful in cancer therapy. Tanveer et al. [31] presented a theoretical investigation on the motion of a nanofluid under electroosmosis and peristalsis. Sharma et al. [32] provided analytical solutions for nanofluid flow in a fluidic peristaltic channel with electroosmosis and their findings can be useful in particle filtrations and cell therapeutics. Prakash et al. [33] studied the motion of a tangent hyperbolic nanofluid via peristaltic pumping with electroosmosis effects with the help of the perturbation method. Noreen et al. [34] discussed the flow of an EMHD nanofluid in an asymmetric microfluidic channel. Mekheimer et al. [35] used gold nanoparticles in a base fluid to discuss the flow characteristics in an EMHD peristaltic channel.

Motivated by the aforementioned investigations, the effect of electroosmosis, magnetic field, activation energy, bioconvection and radiation on the gold–blood nanofluid flow in an asymmetric peristaltic channel is considered in the current article. The different shapes of nanoparticles are considered in the present model since the nanoparticles show characteristic colors and properties with variations in size and shape, which can be utilized in cutting-edge materials, biological imaging and biomedicine [36]. The governing equations are simplified under the lubrication approach and Debye–Hückel linearization process. The resulting highly non-linear system of differential equations area solved numerically under the mathematical computational scheme MATLAB bvp4c. The pictorial representations for the velocity, temperature, nanoparticle volume fraction, shear stress and microorganism concentration are presented for sundry parameters.

## 2. Mathematical Model

### 2.1. Problem Formulation

**Figure 1:**
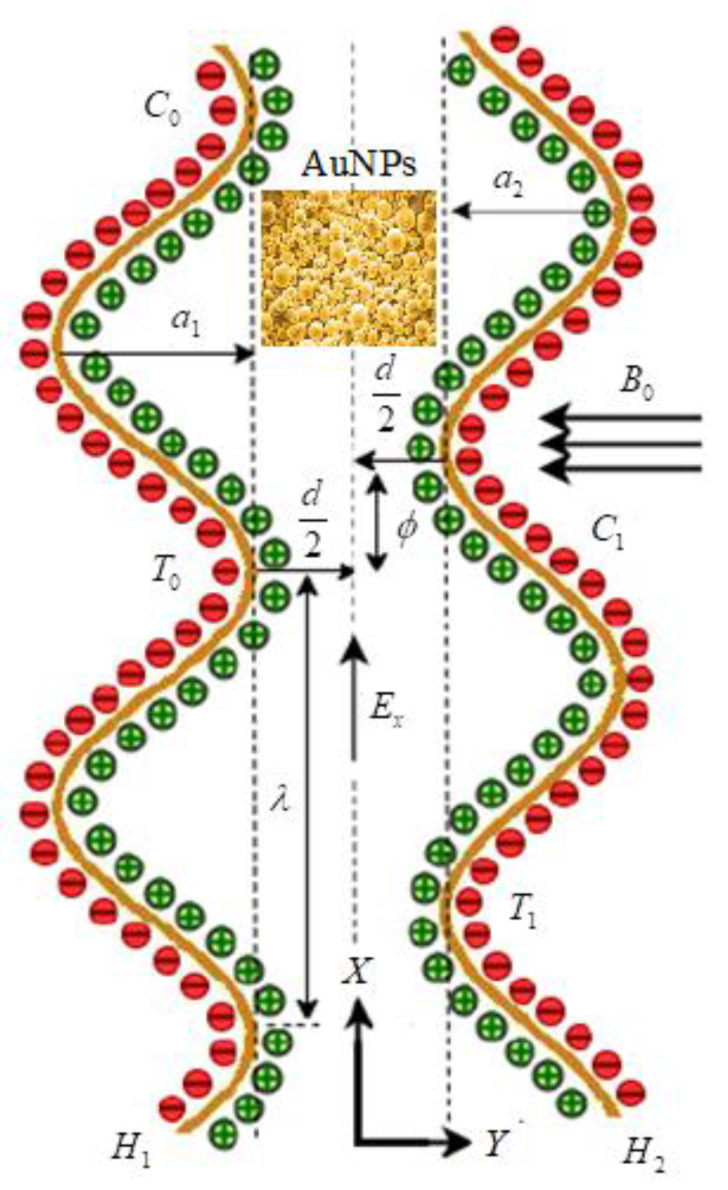
Schematic flow representation in the presence of EMHD and gold nanoparticles.

Peristalsis is a contraction and relaxation of muscles that propagates in a wave down a channel/tube. This kind of situation can be seen in many physiological situations, such as food flow through the esophagus, bile flow through the bile duct and intrauterine fluid motions. For example, in the digestive tract, peristalsis starts in the esophagus where strong wave-like motions of the smooth muscle move balls of swallowed food to the stomach. There, the food is churned into a liquid mixture called chyme that moves into the small intestine where peristalsis continues. We considered the mathematical modeling of EMHD gold–blood nanofluid flow in a peristaltic asymmetric vessel. The influence of bioconvection, activation energy, thermal radiation, and the different shapes of nanoparticles, such as bricks, platelets and cylinders, were taken into account. The Cartesian (X,Y) system is assumed transverse and parallel to the path of wave propagation. The motion is assumed under the electroosmosis and peristalsis behavior. The uniform magnetic field $B_{0}$ is applied in the transverse direction of the actual fluid motion. It is also assumed that the left wall of the peristalsis was maintained at temperature, concentrations and motile organisms as $T_{0},C_{0},N_{0}$. The temperature, concentrations and motile organisms of right peristalsis wall were considered as $T_{1},C_{1},N_{1}\;$(see Figure 1). The geometries of the left and right peristalsis walls were represented as [37]

(1) $$H_{1}\left(X,t\right)=-d-a_{1}\cos^{2}\left(\frac{\pi }{\lambda }\left(X-ct\right)+\phi \right),$$

(2) $$H_{2}\left(X,t\right)=d+a_{2}\cos^{2}\left(\frac{\pi }{\lambda }\left(X-ct\right)\right),$$

where $H_{1}$ is the left peristaltic wall, $H_{2}$ is the right peristaltic wall, d denotes the width of the channel, $a_{1}$ represents wave amplitude of left wall, $a_{2}$ is the wave amplitude of right wall, λ denotes the wavelength, t is the time, c is the wave speed and ϕ represents phase difference; for ϕ=0, the corresponding problem is converted to symmetric model.

The governing equations (continuity, momentum, energy, nanoparticle volume fraction and microorganisms) for the nanofluid flow in the fixed frame are expressed as [38,39,40].

(3) $$\frac{\partial U}{\partial X}+\frac{\partial V}{\partial Y}=0,$$

(4) $$\rho_{nf}\left(\frac{\partial U}{\partial t}+U\frac{\partial U}{\partial X}+V\frac{\partial U}{\partial Y}\right)=-\frac{\partial P}{\partial X}+\mu_{nf}\left(\frac{\partial^{2}U}{\partial X^{2}}+\frac{\partial^{2}U}{\partial Y^{2}}\right)-\sigma_{nf}B_{0}{}^{2}U+\rho_{e}E_{x}\;+{\left(\rho \beta \right)}_{nf}g\left(1-C_{0}\right)\left(T-T_{0}\right)-g\left(\rho_{p}-\rho_{f}\right)\left(C-C_{0}\right)-g\gamma \left(\rho_{m}-\rho_{f}\right)\left(N-N_{0}\right),$$

(5) $$\rho_{nf}\left(\frac{\partial V}{\partial t}+U\frac{\partial V}{\partial X}+V\frac{\partial V}{\partial Y}\right)=-\frac{\partial P}{\partial Y}+\mu_{nf}\left(\frac{\partial^{2}V}{\partial X^{2}}+\frac{\partial^{2}V}{\partial Y^{2}}\right),$$

(6) $${\left(\rho c_{p}\right)}_{nf}\left(\frac{\partial T}{\partial t}+U\frac{\partial T}{\partial X}+V\frac{\partial T}{\partial Y}\right)=\left(k_{nf}+\frac{16\sigma^{*}T_{0}{}^{3}}{3k^{*}}\right)\left(\frac{\partial^{2}T}{\partial X^{2}}+\frac{\partial^{2}T}{\partial Y^{2}}\right)\;+{\left(\rho c_{p}\right)}_{p}\left(D_{B}\left(\frac{\partial C}{\partial X}\frac{\partial T}{\partial X}+\frac{\partial C}{\partial Y}\frac{\partial T}{\partial Y}\right)+\frac{D_{T}}{T_{m}}\left({\left(\frac{\partial T}{\partial X}\right)}^{2}+{\left(\frac{\partial T}{\partial Y}\right)}^{2}\right)\right),$$

(7) $$\frac{\partial C}{\partial t}+U\frac{\partial C}{\partial X}+V\frac{\partial C}{\partial Y}=D_{B}\left(\frac{\partial^{2}C}{\partial X^{2}}+\frac{\partial^{2}C}{\partial Y^{2}}\right)+\frac{D_{T}}{T_{m}}\left(\frac{\partial^{2}T}{\partial X^{2}}+\frac{\partial^{2}T}{\partial Y^{2}}\right)-k_{r}{}^{2}\left(C-C_{0}\right){\left(\frac{T}{T_{0}}\right)}^{n}\exp\left(\frac{-E_{a}}{\omega T}\right),$$

(8) $$\frac{\partial N}{\partial t}+U\frac{\partial N}{\partial X}+V\frac{\partial N}{\partial Y}+\frac{b^{*}W_{e}}{\left(C_{1}-C_{0}\right)}\left(\frac{\partial }{\partial X}\left(N\frac{\partial C}{\partial X}\right)+\frac{\partial }{\partial Y}\left(N\frac{\partial C}{\partial Y}\right)\right)=D_{m}\left(\frac{\partial^{2}N}{\partial X^{2}}+\frac{\partial^{2}N}{\partial Y^{2}}\right),$$

with the corresponding boundary conditions [41,42,43]:

(9) $$U=0,\;\psi =-\frac{F}{2},\;T=T_{0},\;C=C_{0},\;N=N_{0}\;\mathrm{at}\;Y=H_{1}\left(=-d-a_{1}\cos^{2}\left(\frac{\pi }{\lambda }\left(X-ct\right)+\phi \right)\right),$$

(10) $$U=0,\;\psi =\frac{F}{2},\;T=T_{1},\;C=C_{1},\;N=N_{1}\;\mathrm{at}\;Y=H_{2}\left(=d+a_{2}\cos^{2}\left(\frac{\pi }{\lambda }\left(X-ct\right)\right)\right),$$

where U,V denote the velocities in X and Y directions, respectively; $\rho_{nf}$ is nanofluid effective density; t is time; $\mu_{nf}$ is the nanofluid dynamic viscosity; $\sigma_{nf}$ is the nanofluid electrical conductivity; ${\left(\rho \beta \right)}_{nf}$ is the effective thermal expansion; g is the gravitational force; T is the nanoparticle temperature; $\rho_{p}$ is density of nanoparticle; $\rho_{f}$ is density of the base fluid; N is motile density; $N_{0}$ is ambient concentration of motile organisms; γ is volume of microorganisms; $\rho_{m}$ is motile organisms density; $\rho_{e}$ is electrical charge density; $E_{x}$ is applied electric field; ${\left(\rho c_{p}\right)}_{nf}$ is effective heat capacity of nanofluid; $k_{nf}$ is thermal diffusivity of nanofluid; $\sigma^{*}$ is the Stefan–Boltzmann constant; $k^{*}$ is mean absorption coefficient; $D_{B}$ is Brownian diffusion coefficient; C is nanoparticle concentration; $D_{T}$ is thermophoretic diffusion coefficient; $T_{m}$ is mean temperature; $k_{r}$ is the rate of reaction; n is the fitted rate (−1<n<1); $E_{a}$ is activation energy; ω is the Boltzmann constant $\left(8.61\times 10^{-5}ev/K\right)$; $b^{*}$ is chemotaxis constant; $W_{e}$ is swimming cells speed; P is pressure; ψ is the dimensional stream function; F is the dimensional constant flow rate; and $D_{m}$ is the microorganism diffusion coefficient. In Equation (6), the radiation effects were considered under the Rosseland approximation and simplified with the help of Taylor’s series expansion.

### 2.2. Electrohydrodynamics

In a micro-channel, the Poisson equation is defined [44] as:

(11) $$\nabla^{2}\bar{\varphi }=-\frac{\rho_{e}}{\xi },$$

where $\bar{\varphi }$ denotes the electric potential, ξ is the dielectric permittivity, and $\rho_{e}\;$represents the total charge density. 

The net charge density $\rho_{e}$ follows the Boltzmann variation [45] and is

(12) $$\rho_{e}=-z_{v}e\left({\bar{n}}^{-}-{\bar{n}}^{+}\right).$$

The anions (${\bar{n}}^{-}$) and captions (¯${\bar{n}}^{+}$) are distinct by $\rho_{e}$ of the Boltzmann equation:

(13) $${\bar{n}}^{\pm }=n_{0}e^{\left(\pm \frac{ez_{v}}{T_{av}K_{B}}\bar{\varphi }\right)},$$

where, $n_{0}$ denotes bulk concentration, $K_{B}$ the Boltzmann constant, $z_{v}$ the charge balance, e the electronic charge and $T_{av}$ is the average temperature. Employing Debye–Hückel linearization estimation [45], Equation (11) revolves to

(14) $$\frac{d^{2}\varphi }{dy^{2}}=\kappa^{2}\varphi ,$$

where κ represents the electroosmotic expression. The analytical solution of Equation (14) with boundary conditions (BCs): $\varphi =\zeta_{1}$ at $y=h_{1}$ and $\varphi =\zeta_{2}$ at $y=h_{2}$ was reached as:

(15) $$\varphi =\left(\frac{\varsigma_{2}\sinh\left({\kappa h}_{1}\right)-\varsigma_{1}\sinh\left({\kappa h}_{2}\right)}{\sinh\left(\kappa \left(h_{1}-h_{2}\right)\right)}\right)\cosh\left(\kappa y\right)-\left(\frac{\varsigma_{2}\cosh\left({\kappa h}_{1}\right)-\varsigma_{1}\cosh\left({\kappa h}_{2}\right)}{\sinh\left(\kappa \left(h_{1}-h_{2}\right)\right)}\right)\sinh\left(\kappa y\right).$$

### 2.3. Thermophysical Properties and Geometries of Nanoparticles

The thermophysical characteristics of gold blood nanofluid are given in Table 1, and are expressed via equations as follow [41,46,47]:

(16) $$\rho_{nf}=\rho_{f}\left(\left(1-\varphi_{1}\right)+\varphi_{1}\left(\frac{\rho_{p}}{\rho_{s}}\right)\right),$$

(17) $${\left(\rho \beta \right)}_{nf}={\left(\rho \beta \right)}_{f}\left(\left(1-\varphi_{1}\right)+\varphi_{1}\left(\frac{{\left(\rho \beta \right)}_{p}}{{\left(\rho \beta \right)}_{f}}\right)\right),$$

(18) $${\left(\rho c_{p}\right)}_{nf}={\left(\rho c_{p}\right)}_{f}\left(\left(1-\varphi_{1}\right)+\varphi_{1}\left(\frac{{\left(\rho c_{p}\right)}_{p}}{{\left(\rho c_{p}\right)}_{f}}\right)\right),$$

(19) $$\mu_{nf}=\mu_{f}\left(1+A_{1}\varphi_{1}+A_{2}\varphi_{1}^{2}\right),$$

(20) $$k_{nf}=k_{f}\left(\frac{k_{p}+\left(s-1\right)k_{f}-\left(s-1\right)\varphi_{1}\left(k_{f}-k_{p}\right)}{k_{p}+\left(s-1\right)k_{f}+\varphi_{1}\left(k_{f}-k_{p}\right)}\right),$$

(21) $$\sigma_{nf}=\sigma_{f}\left(1+\frac{3\left(\frac{\sigma_{p}}{\sigma_{f}}-1\right)\varphi_{1}}{\left(\frac{\sigma_{p}}{\sigma_{f}}+2\right)-\left(\frac{\sigma_{p}}{\sigma_{f}}-1\right)\varphi_{1}}\right),$$

k(W/mK) $c_{p}\left(J/kgK\right)$ $\rho \left(kg/m^{3}\right)$ σ(S/m) $\beta \left(1/k\right)\times 10^{-5}$ (kg/m.s)

### 2.4. Non-Dimensional Governing Equations and Boundary Conditions

We introduce the transformations between the wave and fixed frame:

(22) $$x=X-ct,\;\;y=Y,\;\;u=U-c,\;\;v=V,\;\;p=P,\bar{\;N}=N,\;\;\bar{T}=T,\bar{\;C}=C.$$

Additionally, the non-dimensional quantities were:

(23) u¯=uc, v¯=vcδ, x¯=xλ,y¯=yd,p¯=d2pcλμf,θ=T¯−T0T1−T0,σ=C¯−C0C1−C0,χ=N¯−N0N1−N0,M=σfμfB0d,Re=ρfcdμf,δ=dλ,Rb=(ρm−ρf)γ(N1−N0)(ρβ)f(1−C0)(T1−T0),Gr=g(ρβ)f(1−C0)(T1−T0)d2cμf,Nr=(ρm−ρf)(C1−C0)(ρβ)f(1−C0)(T1−T0),Rn=16σ*T033k*μf(cp)f,τ=(ρcp)p(ρcp)f,Pr=μf(cp)fkf,ξ=kr2d2μf,Sc=μfρfDB,β=T1−T0T0,E=EaωT0,Nb=ρfτDB(C1−C0)μf,Nt=ρfτDT(T1−T0)μfTm,Pe=b1*WeDm,Ω=N0N1−N0,UHS=−Exεefξ1cμf,κ=dez2n0εefkBTe,u=∂ψ∂y,v=−δ∂ψ∂x,ψ¯=ψca1,F¯=Fcd .

The analysis was carried out under the assumption that the width of the channel is small compared to the wavelength of peristaltic waves. This assumption is usually called long wavelength approximation. Such consideration is realistic when peristalsis for ureter, chyme movement in intestine and spermatozoa in ductus efferences are considered. The Reynolds number was taken low. The long wavelength and low Reynolds number approximations are used extensively in the analysis of peristaltic flows. It should be pointed out that the theory of long wavelength and zero Reynolds number remains applicable for case of chyme transport in small intestine [48].

Using the aforementioned theory, and assumptions (long wavelength and low Reynolds number), the appropriate non-dimensional governing equations (in wave frame) are written as:

(24) $$\frac{\mu_{nf}}{\mu_{f}}\frac{\partial^{4}\psi }{\partial y^{4}}-\frac{\sigma_{nf}}{\sigma_{f}}M^{2}\frac{\partial^{2}\psi }{\partial y^{2}}+Gr\left(\frac{{\left(\rho \beta \right)}_{nf}}{{\left(\rho \beta \right)}_{f}}\frac{\partial \theta }{\partial y}-Nr\frac{\partial \sigma }{\partial y}-Rb\frac{\partial \chi }{\partial y}\right)\;+\kappa^{3}U_{HS}\left(\left(\frac{\varsigma_{2}\sinh\left(\kappa h_{1}\right)-\varsigma_{1}\sinh\left(\kappa h_{2}\right)}{\sinh\left(\kappa \left(h_{1}-h_{2}\right)\right)}\right)\sinh\left(\kappa y\right)\;-\left(\frac{\varsigma_{2}\cosh\left(\kappa h_{1}\right)-\varsigma_{1}\cosh\left(\kappa h_{2}\right)}{\sinh\left(\kappa \left(h_{1}-h_{2}\right)\right)}\right)\cosh\left(\kappa y\right)\right)=0,$$

(25) $$\left(\frac{k_{nf}}{k_{f}}+RnPr\right)\frac{\partial^{2}\theta }{\partial y^{2}}+Nb\;Pr\frac{\partial \theta }{\partial y}\frac{\partial \sigma }{\partial y}+Nt\;Pr{\left(\frac{\partial \theta }{\partial y}\right)}^{2}=0\;,$$

(26) $$\frac{\partial^{2}\sigma }{\partial y^{2}}+\frac{Nt}{Nb}\frac{\partial^{2}\theta }{\partial y^{2}}-Sc\xi \sigma {\left(1+\beta \theta \right)}^{n}\exp\left(-\frac{E}{1+\beta \theta }\right)=0,$$

(27) $$\frac{\partial^{2}\chi }{\partial y^{2}}-Pe\left(\chi +\Omega \right)\frac{\partial^{2}\sigma }{\partial y^{2}}-Pe\frac{\partial \chi }{\partial y}\frac{\partial \sigma }{\partial y}=0,$$

with the corresponding dimensionless boundary conditions

(28) $$\psi =-\frac{F}{2},\;\frac{\partial \psi }{\partial y}=-1,\;\theta =0,\sigma =0,\;\chi =0\;\;\mathrm{at}\;y=h_{1}\left(=-1-a\cos^{2}\left(\pi x+\phi \right)\right),$$

(29) $$\psi =\frac{F}{2},\;\frac{\partial \psi }{\partial y}=-1,\;\theta =1,\;\sigma =1,\;\chi =1\;\;\mathrm{at}\;y=h_{2}\left(=1+b\cos^{2}\left(\pi x\right)\right),$$

where Q(=F+2+(a+b)/2) is the time mean flow rate in the fixed frame and $F=\int_{h_{1}}^{h_{2}}\left(\partial \psi /\partial y\right)dy$ is the time mean flow rate in the wave frame.

In the above expressions, θ is nanoparticle temperature; σ is nanoparticle concentration; χ is motile microorganisms; M is Hartmann number; Re is the Reynolds number; δ is the wave number; Rb is the bioconvection Rayleigh constant; Gr is the thermal Grashof number; Nr is the buoyancy ratio constant; Rn is the radiation parameter; τ is the effective heat capacity ratio of nanoparticle material-to-liquid heat capacity; Pr is the Prandtl number; ξ is the reaction rate constant; Sc is the Schmidt number; β is the temperature ratio parameter; E is the activation energy parameter; Nb is the Brownian motion parameter; Nt is the thermophoresis parameter; Pe is the Peclet number; Ω is the concentration difference constant for the microorganisms; $U_{HS}$ is the Helmholtz–Smoluchowski velocity; κ is the electroosmosis parameter; $\varsigma_{1}$ and $\varsigma_{2}$ are the zeta potentials; F is the volume flow rate in the wave frame; and ψ is stream function.

The standard non-dimensional shear stress at the left wall can be represented as

(30) $$\tau_{s}=\frac{\mu_{nf}}{\mu_{f}}{\left(\frac{\partial u}{\partial y}\right)}_{y=h_{1}}.$$

## 3. Numerical Procedure

The numerical solution of Equations (24)–(27) along with (28)–(29) can be represented with bvp4c scheme. The mechanism behind bvp4c MATLAB software is the finite difference method. The system of higher order differential equations is renovated into an ordinary differential equation, representing $\psi =y_{1},\;\psi^{\prime }=y_{2},\;\psi^{\prime \prime }=y_{3},\;\psi^{\prime \prime \prime }=y_{4},\;\theta =y_{5},\;\theta^{\prime }=y_{6},\;\sigma =y_{7},\;\sigma^{\prime }=y_{8},\;\chi =y_{9},\;\chi^{\prime }=y_{10}.\;$Thus Equations (24)–(29) can be expressed as

(31) $$\begin{array}{l} y_{1}^{\prime }=y_{2},\\ y_{2}^{\prime }=y_{3},\\ y_{3}^{\prime }=y_{4},\\ y_{4}^{\prime }=\frac{1}{a_{1}}(\begin{array}{l} a_{2}M^{2}y_{3}-Gr(a_{3}y_{6}-Nry_{8}-Rby_{10})\\ -\kappa^{3}U_{HS}\left(\left(\frac{\varsigma_{2}\sinh\left(\kappa h_{1}\right)-\varsigma_{1}\sinh\left(\kappa h_{2}\right)}{\sinh\left(\kappa \left(h_{1}-h_{2}\right)\right)}\right)\sinh\left(\kappa y\right)-\left(\frac{\varsigma_{2}\cosh\left(\kappa h_{1}\right)-\varsigma_{1}\cosh\left(\kappa h_{2}\right)}{\sinh\left(\kappa \left(h_{1}-h_{2}\right)\right)}\right)\cosh\left(\kappa y\right)\right) \end{array}),\\ y_{5}^{\prime }=y_{6},\\ y_{6}^{\prime }=\frac{-1}{a_{4}+RnPr}\left(Nb\mathrm{Pr}y_{6}y_{8}+Nt\mathrm{Pr}{\left(y_{6}\right)}^{2}\right)\\ y_{7}^{\prime }=y_{8},\\ y_{8}^{\prime }=-\frac{Nt}{Nb}y_{6}^{\prime }+Sc\xi y_{7}{\left(1+\beta y_{5}\right)}^{n}\exp\left(-\frac{E}{1+\beta y_{5}}\right),\\ y_{9}^{\prime }=y_{10},\\ y_{10}^{\prime }=Pe\left(\chi +\Omega \right)y_{8}^{\prime }+Pey_{8}y_{10} \end{array}$$

with the following boundary conditions:

(32) $$y_{1}\left(h_{1}\right)=-\frac{F}{2},\;y_{2}\left(h_{1}\right)=-1,\;y_{5}\left(h_{1}\right)=0,\;y_{7}\left(h_{1}\right)=0,\;y_{9}\left(h_{1}\right)=0;\;y_{1}\left(h_{2}\right)=\frac{F}{2},\;y_{2}\left(h_{2}\right)=-1,\;y_{5}\left(h_{2}\right)=1,\;y_{7}\left(h_{2}\right)=1,\;y_{9}\left(h_{2}\right)=1.$$

## 4. Results and Discussion

**Figure 2:**
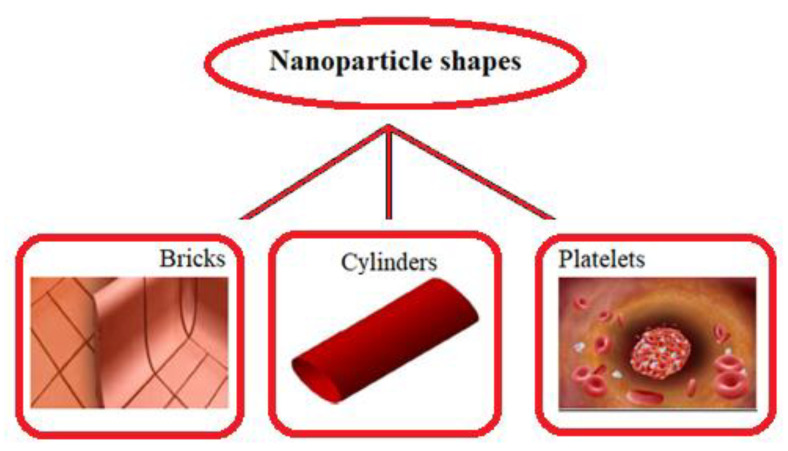
Different nanoparticle shapes.

**Figure 3:**
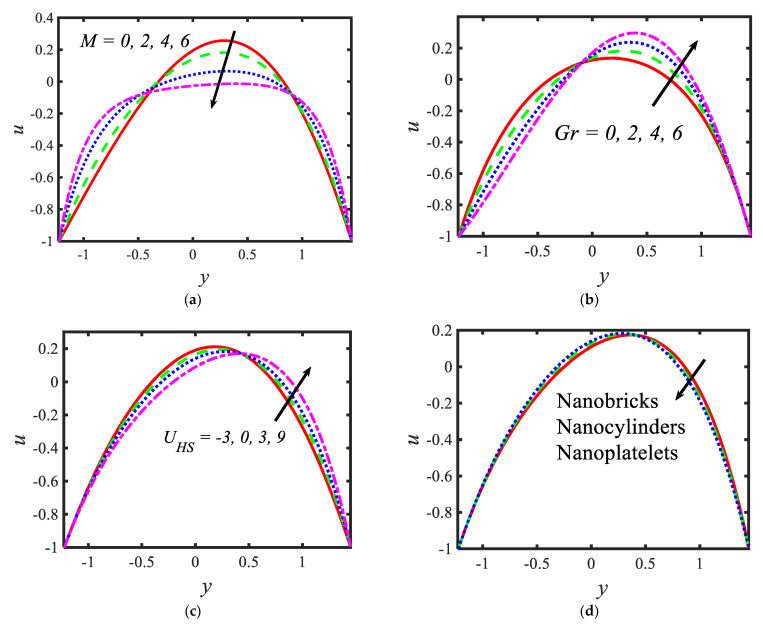
Variations in the velocity profiles for (**a**) the Hartmann number, (**b**) Grashof number, (**c**) Helmholtz–Smoluchowski velocity and (**d**) shape factor.

In the current section, pictorial representations are presented for the velocity u, temperature θ, nanoparticle volume fraction σ, microorganisms χ, shear stress $\tau_{s}$ and Nusselt number Nu for the various parameters with various ranges, such as the Hartmann number M(range 0−6); thermal Grashof number M(range 0−6); Helmholtz–Smoluchowski velocity $U_{HS}\left(\mathrm{range}\;-3-9\right)$; shape factor s (nanoparticle shapes are provided in Figure 2, and the values of the shape factors can be found in Table 2); radiation parameter Rn(range 0.1−0.7); thermophoresis parameter Nt(range 1−9); Brownian motion parameter Nb(range 0.1−0.9); activation energy parameter E(range 1−4); temperature ratio parameter β(range 0−3); Peclet number Pe(range 0−0.9); electroosmosis parameter κ(range 2−2.3); zeta potential $\varsigma_{1}\left(\mathrm{range}\;1-1.6\right)$; bioconvection Rayleigh constant Rb(range 1−1.6) and buoyancy ratio constant Nr(range 1−1.6). Figure 3 presents the influences of various parameters, such as the Hartmann number M, thermal Grashof number Gr, Helmholtz–Smoluchowski velocity $U_{HS}$ and the shape factor s on the velocity distribution. It can be concluded from Figure 3 that the profiles are parabolic in nature and the highest velocities are noticed in the middle portion of the peristaltic waves. The velocity reduction is noticed with higher values of the Hartmann number in the middle part of the channel and the trend is reversed near the walls (see Figure 3a). In the presence of a magnetic field, a force called the Lorentz force arises in the flow domain and it leads to a reduction in the fluid flow rate. Moreover, larger velocities are noticed in the absence of the magnetic field. It can be observed in Figure 3b that, with the increasing values of the thermal Grashof number, the velocity of the nanofluid rises near the right wall and reduces near the left wall. It can also be determined that buoyancy forces play a significant role in peristaltic movement. They support the fluid flow along the microchannel. The trend is reversed in case of the Helmholtz–Smoluchowski velocity (see Figure 3c). Figure 3d represents the effects of various nanoparticle shapes, such as bricks, cylinders and platelets, on the velocity profile. It can be seen that the larger velocities are observed in the flow of the nanofluid containing brick-shaped nanoparticles near the right wall, and later the sequence is followed as cylindrical and platelet-shaped nanoparticles, respectively. 

**Figure 4:**
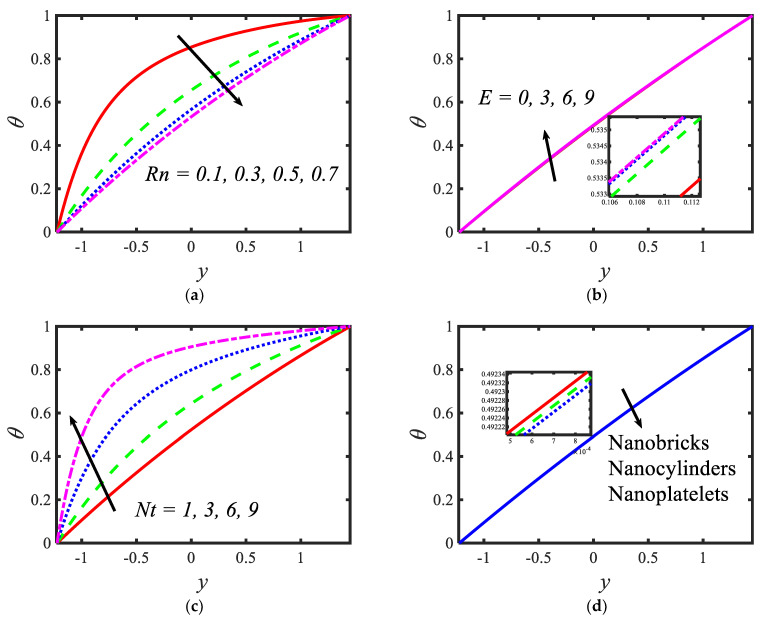
Variations in temperature profiles for (**a**) the radiation parameter, (**b**) Prandtl number, (**c**) thermophoresis parameter and (**d**) shape factor.

**Figure 5:**
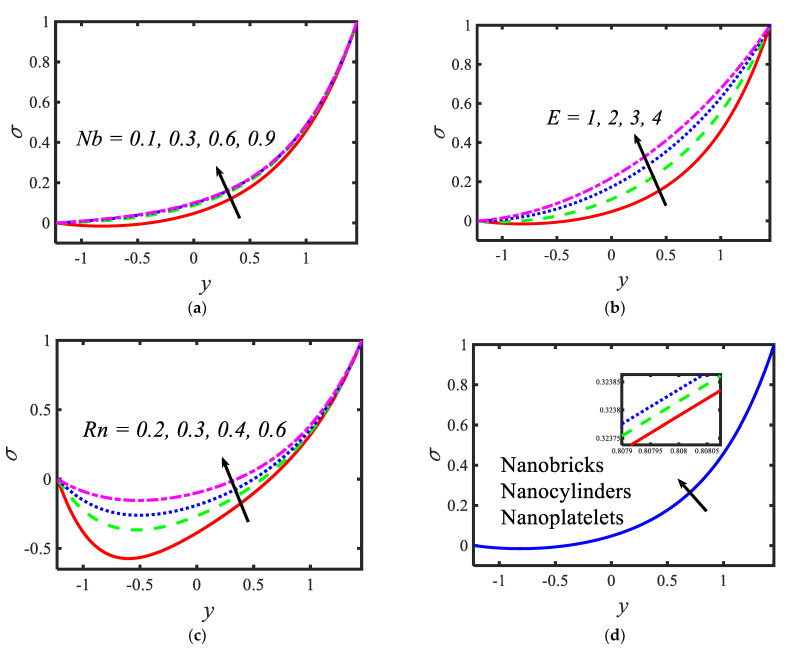
Variations in nanoparticle volume fraction profiles for (**a**) the Brownian motion parameter, (**b**) activation energy parameter, (**c**) radiation parameter and (**d**) the Shape factor.

Figure 4 presents the variations in the temperatures for various values of the radiation parameter Rn , activation energy E, thermophoresis parameter Nt and shape factor s. From the temperature profiles, it can be observed that the structure is likely to be perceived as parabolic in nature. It is clear from Figure 4a that the temperature decreases with the rising values of the radiation parameter. Activation energy increases the temperature (see Figure 4b). A lower thermophoresis parameter produces a lower temperature, and a higher thermophoresis parameter produces a higher temperature (see Figure 4c). Figure 4d depicts the temperature profile for different nanoparticle shapes. It determines that a higher temperature is noticed in the nanofluid flow suspended by brick-shaped nanoparticles and a lower temperature is depicted for platelet-shaped nanoparticles. Figure 5 presents the behavior of the nanoparticle volume fraction with different parameters, for instance, the Brownian motion parameter Nb , activation energy parameter E, radiation parameter Rn and shape factor s. It is clear from Figure 5a that the nanoparticle volume fraction increases with the rising values of the Brownian motion parameter. It can be observed that the nanoparticle volume fraction enhances the activation energy (see Figure 5b). From Figure 5c, it can be observed that as the radiation parameter increases, the nanoparticle volume fraction increases. Figure 5d shows the nanoparticle volume fraction with different nanoparticle shapes in the nanofluid. A lower nanoparticle volume fraction is observed for the nanofluid flow with brick-shaped nanoparticles in the nanofluid, and a higher nanoparticle volume fraction is seen in the case of platelet-shaped nanoparticles in the nanofluid.

**Figure 6:**
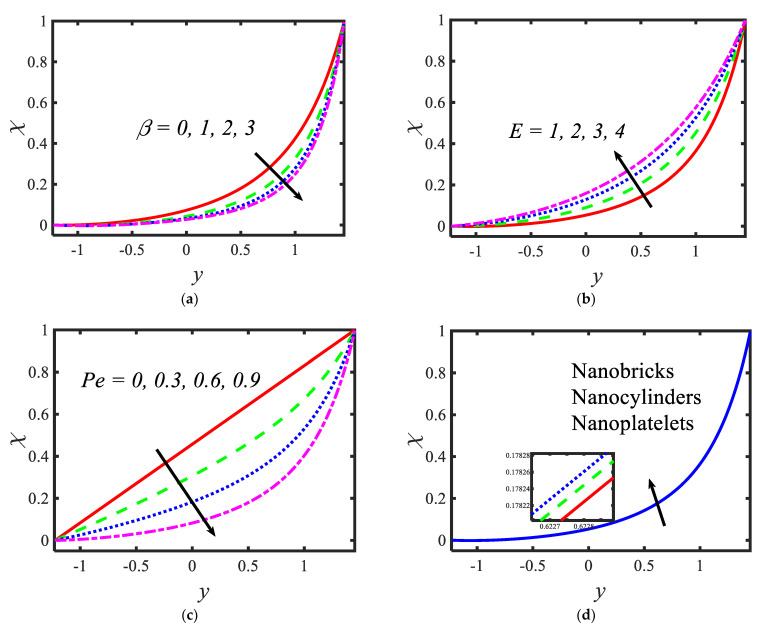
Variations in the motile microorganism profile for (**a**) the temperature ratio parameter, (**b**) activation energy parameter, (**c**) Prandtl number and (**d**) shape factor.

**Figure 7:**
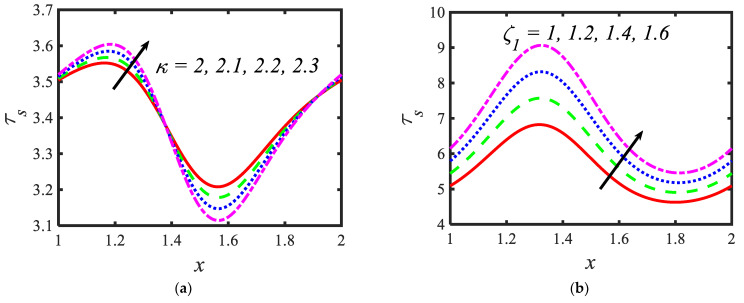

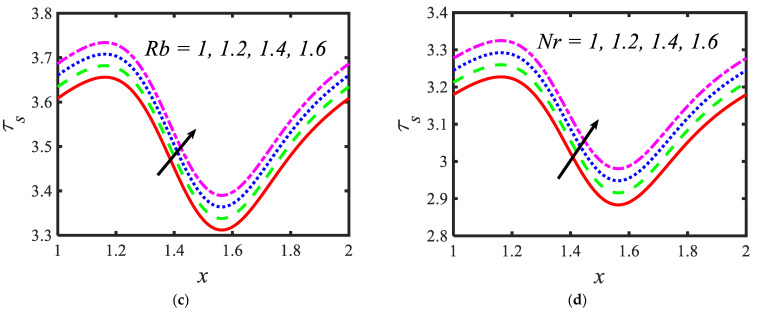
Variations in the shear stress profiles for (**a**) the electroosmosis parameter, (**b**) zeta potential, (**c**) bioconvection Rayleigh constant and (**d**) buoyancy ratio constant.

**Figure 8:**
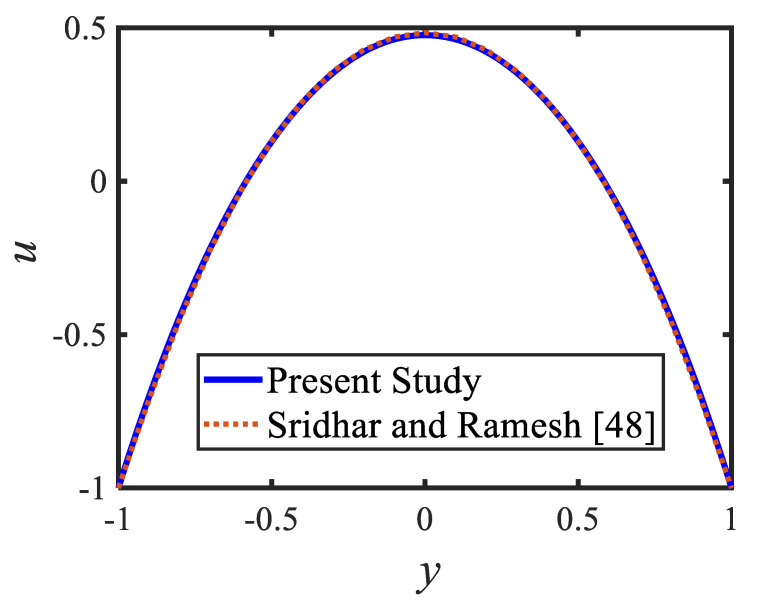
Comparison of the velocity profiles of the present study and [51].

Figure 6 is plotted to judge the variation of microorganism concentrations for various parameters, such as activation energy parameter E, temperature ratio parameter β, Peclet number Pe and shape factor s. It is clear from these figures that the behavior of the microorganism concentration is parabolic. From Figure 6a, it can be observed that the motile microorganisms reduce in the channel of the peristalsis with the rising values of the temperature ratio parameter. Activation energy increases the microorganism concentration profile in the peristaltic flow. Microorganism concentration is a decreasing function of the Peclet number (see Figure 6c). In Figure 6d, it can be observed that the platelet-shaped nanoparticles involved in the nanofluid present the highest microorganism concentration, and the brick-shaped nanoparticles involved in the nanofluid present the lowest concentration. Figure 7 presents the effects of the electroosmosis parameter, zeta potential, bioconvection Rayleigh constant and buoyancy ratio constant on the shear stress distribution. Shear stress distribution provides a wave-like form due to peristalsis. It is noted from Figure 7a that the electroosmosis parameter displays the mixed behavior in the channel. Moreover, it is observed from Figure 7b–d that the shear stress increases with the increasing values of the zeta potential, bioconvection Rayleigh constant and buoyancy ratio constant. The present study is in good agreement with the existing literature by Sridhar and Ramesh [50] (see Figure 8).

## 5. Conclusions

In the current article, the influence of activation energy and bioconvection on the propulsion of gold–blood nanofluid is considered in an asymmetric peristaltic channel. The effects of the magnetic field, radiation and electroosmosis were also considered. The system of non-dimensional highly non-linear differential equations were solved by utilizing computational software MATLAB with the help of the bvp4c function. The graphical results were presented for the velocity, temperature, nanoparticle volume fraction, microorganism concentration and shear stress with respect to the sundry parameters. The main findings of the current study are the following:

- The velocity of nanofluid flow suspended by brick-shaped nanoparticles is higher near the right wall, compared with platelet= and cylinder-shaped nanoparticles.
- A higher magnetic field strength suppresses the velocity of the nanofluid.
- Temperature is an increasing function of activation energy and the thermophoresis parameter.
- The radiation parameter reduces the temperature profile.
- The nanoparticle volume fraction increases with the rising values of the Brownian motion parameter and radiation parameter.
- Microorganism concentration increases with the rising values of activation energy.
- Shear stress is an increasing function of zeta potentials and the bioconvection Rayleigh constant.

## Figures and Tables

**Table 1:** Thermophysical properties of gold nanoparticles and base fluid (blood) [43,47].

Properties	Gold	Blood
k(W/mK)	314	0.492
$c_{p}\left(J/kgK\right)$	129	3594
$\rho \left(kg/m^{3}\right)$	19,320	1063
σ(S/m)	$4.52\times 10^{7}$	0.667
$\beta \left(1/k\right)\times 10^{-5}$	1.4	0.18
(kg/m.s)	-	0.0004
Pr	-	21

**Table 2:** Shape factors and coefficients of the nanoparticles [47,49].

Nanoparticle Shapes	Shape Factor (s)	$A_{1}$	$A_{2}$
Bricks	3.72	1.9	471.4
Platelets	5.72	37.1	612.6
Cylinders	4.82	13.5	904.4

## Data Availability

The data that support the findings of this study are available from the corresponding author upon reasonable request.

## Nomenclature

(X,Y)	axial and transverse directions of the flow
$B_{0}$	uniform magnetic field
$T_{0}$	temperature at left wall
$C_{0}$	concentration at left wall
$N_{0}$	motile organism concentration at left wall
$T_{1}$	temperature at right wall
$C_{1}$	concentration at right wall
$N_{1}$	motile organism concentration at right wall
$H_{1}$	left peristaltic wall
$H_{2}$	right peristaltic wall
d	width of the channel
$a_{1}$	wave amplitude of left wall
$a_{2}$	wave amplitude of right wall
λ	wavelength
t	time
c	wave speed
ϕ	phase difference
U,V	velocities in X and Y directions, respectively
$\rho_{nf}$	nanofluid effective density
$\mu_{nf}$	nanofluid dynamic viscosity
$\sigma_{nf}$	nanofluid electrical conductivity
${\left(\rho \beta \right)}_{nf}$	effective thermal expansion
g	gravitational force
T	nanoparticle temperature
$\rho_{p}$	density of nanoparticles $\rho_{p}$
$\rho_{f}$	density of base fluid
N	motile density
$N_{0}$	ambient concentration of motile organisms
γ	volume of microorganisms
$\rho_{m}$	motile organism density
$\rho_{e}$	electrical charge density
$E_{x}$	applied electric field
${\left(\rho c_{p}\right)}_{nf}$	effective heat capacity of nanofluid
$k_{nf}$	thermal diffusivity of nanofluid
$\sigma^{*}$	Stefan–Boltzmann constant
$k^{*}$	mean absorption coefficient
$D_{B}$	Brownian diffusion coefficient
C	nanoparticle concentration
$D_{T}$	thermophoretic diffusion coefficient
$T_{m}$	mean temperature
$k_{r}$	rate of reaction
n	fitted rate
$E_{a}$	activation energy
ω	Boltzmann constant
$b^{*}$	chemotaxis constant
$W_{e}$	swimming cells speed
P	pressure
ψ	dimensional stream function
F	dimensional constant flow rate
$D_{m}$	microorganism diffusion coefficient
θ	nanoparticle temperature
σ	nanoparticle concentration
χ	motile microorganisms
M	Hartmann number
Re	Reynolds number
δ	wave number
Rb	bioconvection Rayleigh constant
Gr	thermal Grashof number
Nr	buoyancy ratio constant
Rn	radiation parameter
τ	effective heat capacity ratio of nanoparticle material-to-liquid heat capacity
Pr	Prandtl number
ξ	reaction rate constant
Sc	Schmidt number
β	temperature ratio parameter
E	activation energy parameter
Nb	Brownian motion parameter
Nt	thermophoresis parameter
Pe	Peclet number
Ω	concentration difference constant for microorganisms
$U_{HS}$	Helmholtz–Smoluchowski velocity
κ	electroosmosis parameter
$\varsigma_{1},\varsigma_{2}$	zeta potentials
